# The Rise in Tubular pH during Hypercalciuria Exacerbates Calcium Stone Formation

**DOI:** 10.3390/ijms25094787

**Published:** 2024-04-27

**Authors:** Farai C. Gombedza, Samuel Shin, Jaclyn Sadiua, George B. Stackhouse, Bidhan C. Bandyopadhyay

**Affiliations:** 1 Calcium Signaling Laboratory, Research Service, Veterans Affairs Medical Center, 50 Irving Street, NW, Washington, DC 20422, USA; fgombedz@pnw.edu (F.C.G.); samuel.shin@va.gov (S.S.); sadiuajm@alumni.vcu.edu (J.S.); 2 Department of Biomedical Engineering, The Catholic University of America, 620 Michigan Avenue NE, Washington, DC 20064, USA; 3 Urology Service, Veterans Affairs Medical Center, 50 Irving Street, NW, Washington, DC 20422, USA; george.stackhouse@va.gov; 4 Division of Renal Diseases & Hypertension, Department of Medicine, The George Washington University, Washington, DC 20037, USA

**Keywords:** hypercalciuria, renal tubular pH, proximal tubule, oxidative stress, inflammation, fibrosis, apoptosis, calcium nephrolithiasis

## Abstract

In calcium nephrolithiasis (CaNL), most calcium kidney stones are identified as calcium oxalate (CaOx) with variable amounts of calcium phosphate (CaP), where CaP is found as the core component. The nucleation of CaP could be the first step of CaP+CaOx (mixed) stone formation. High urinary supersaturation of CaP due to hypercalciuria and an elevated urine pH have been described as the two main factors in the nucleation of CaP crystals. Our previous in vivo findings (in mice) show that transient receptor potential canonical type 3 (TRPC3)-mediated Ca^2+^ entry triggers a transepithelial Ca^2+^ flux to regulate proximal tubular (PT) luminal [Ca^2+^], and TRPC3-knockout (KO; -/-) mice exhibited moderate hypercalciuria and microcrystal formation at the loop of Henle (LOH). Therefore, we utilized TRPC3 KO mice and exposed them to both hypercalciuric [2% calcium gluconate (CaG) treatment] and alkalineuric conditions [0.08% acetazolamide (ACZ) treatment] to generate a CaNL phenotype. Our results revealed a significant CaP and mixed crystal formation in those treated KO mice (KOT) compared to their WT counterparts (WTT). Importantly, prolonged exposure to CaG and ACZ resulted in a further increase in crystal size for both treated groups (WTT and KOT), but the KOT mice crystal sizes were markedly larger. Moreover, kidney tissue sections of the KOT mice displayed a greater CaP and mixed microcrystal formation than the kidney sections of the WTT group, specifically in the outer and inner medullary and calyceal region; thus, a higher degree of calcifications and mixed calcium lithiasis in the kidneys of the KOT group was displayed. In our effort to find the Ca^2+^ signaling pathophysiology of PT cells, we found that PT cells from both treated groups (WTT and KOT) elicited a larger Ca^2+^ entry compared to the WT counterparts because of significant inhibition by the store-operated Ca^2+^ entry (SOCE) inhibitor, Pyr6. In the presence of both SOCE (Pyr6) and ROCE (receptor-operated Ca^2+^ entry) inhibitors (Pyr10), Ca^2+^ entry by WTT cells was moderately inhibited, suggesting that the Ca^2+^ and pH levels exerted sensitivity changes in response to ROCE and SOCE. An assessment of the gene expression profiles in the PT cells of WTT and KOT mice revealed a safeguarding effect of TRPC3 against detrimental processes (calcification, fibrosis, inflammation, and apoptosis) in the presence of higher pH and hypercalciuric conditions in mice. Together, these findings show that compromise in both the ROCE and SOCE mechanisms in the absence of TRPC3 under hypercalciuric plus higher tubular pH conditions results in higher CaP and mixed crystal formation and that TRPC3 is protective against those adverse effects.

## 1. Introduction

The prevalence of calcium phosphate (CaP) renal stones is increasing, with a high rate of reoccurrence [1]. This condition is caused by high urinary supersaturation of CaP, which arises from genetic hypercalciuria and an elevated tubular urine pH [2]. The rise in urine pH can be caused by renal damage from stone crystals or procedures or treatments for stones [3]. It is also possible that the urine pH may be intrinsically high in some people who are thereby predisposed to CaP stones [4]. Therefore, understanding the underlying mechanisms for CaP formation is crucial.

The proximal tubule (PT) is the site where most of the Ca^2+^ reabsorption takes place, accounting for ~65–70% of the Ca^2+^ being reabsorbed [5]. Previous studies have shown that Ca^2+^ reabsorption from the PT has been implicated in hypercalciuria in calcium kidney stone formers [6,7], highlighting the importance of the role of PT in regulating Ca^2+^ in the nephron tubule, which may prevent downstream saturation for favorable calcium precipitation, possibly at the tip of the loop of Henle (LOH), where the luminal pH normally increases to 7.3 [8]. Most kidney stones (~80%) are identified as mixed calcium stones, which are mostly composed of calcium oxalate (CaOx) with variable amounts of CaP [8]. However, stones with a large composition of CaP were frequently observed in recurrent CaOx stone formers, showing the importance of CaP as the nidus of calcium stone formation [9]. Interestingly, stone formation is not associated with increased rates of chronic kidney disease (CKD) or hypertension, and the majority of calcium stones are formed by idiopathic stone formers that have one or more metabolic abnormalities with no systemic illness [10]. One of those metabolic conditions, hypercalciuria, is considered to be the most common metabolic risk factor for calcium stone formation [9]. Meanwhile, renal tubular acidosis (RTA), which is characterized as defective renal acid-base imbalance due to the inability to secrete H^+^-atoms, may promote CaP formation in the RT [11].

Previous research reports a Ca^2+^-sensing receptor (CaSR) -transient receptor potential canonical (TRPC) 3 complex that triggers a Ca^2+^ entry and leads to a transepithelial Ca^2+^ flux in the mice PT cell [12]. We have also shown that the TRPC3-knockout (KO) mice had a phenotype of elevated urinary Ca^2+^ with CaP crystals in the loop of Henle (LOH), which exhibited moderate hypercalciuria [12]. Prolonged hypercalciuric stress condition in the PT from calcium gluconate (CaG)-treated mice led to prolonged intracellular Ca^2+^ entry, oxidative damage, cellular injury, and various pathophysiological conditions such as inflammation, fibrosis, and/or apoptosis [13]. Furthermore, we found that in treated KO mice, the mode of the Ca^2+^ signaling signature in the PT switched from receptor-operated Ca^2+^ entry (ROCE) to (store-operated Ca^2+^ entry) SOCE due to renal alkalization, which may be linked to significantly enhanced bicarbonate currents to induce CaP crystal formation [13]. More importantly, the renal alkalization in TRPC3 KO mice intensified the pathophysiologic pathway activation of PT cell injury through reactive oxygen species (ROS) generation, calcification, inflammation, fibrosis, and apoptosis [11].

In this study, we investigate the effect of prolonged hypercalciuric stress using alkaline urine in TRPC3 KO mice. With the combination of renal Ca^2+^ and H^+^ atom wasting, we expect mixed crystal formation with upregulated markers for ROS generation, calcification, inflammation, fibrosis, and apoptosis in these mice, which may mimic clinical CKD in stone formers.

## 2. Results

### 2.1. Hypercalciuria in Combination with Alkaline Urine Results in Larger CaP + CaOx Crystal Formation in the Absence of TRPC3

**Figure 1:**
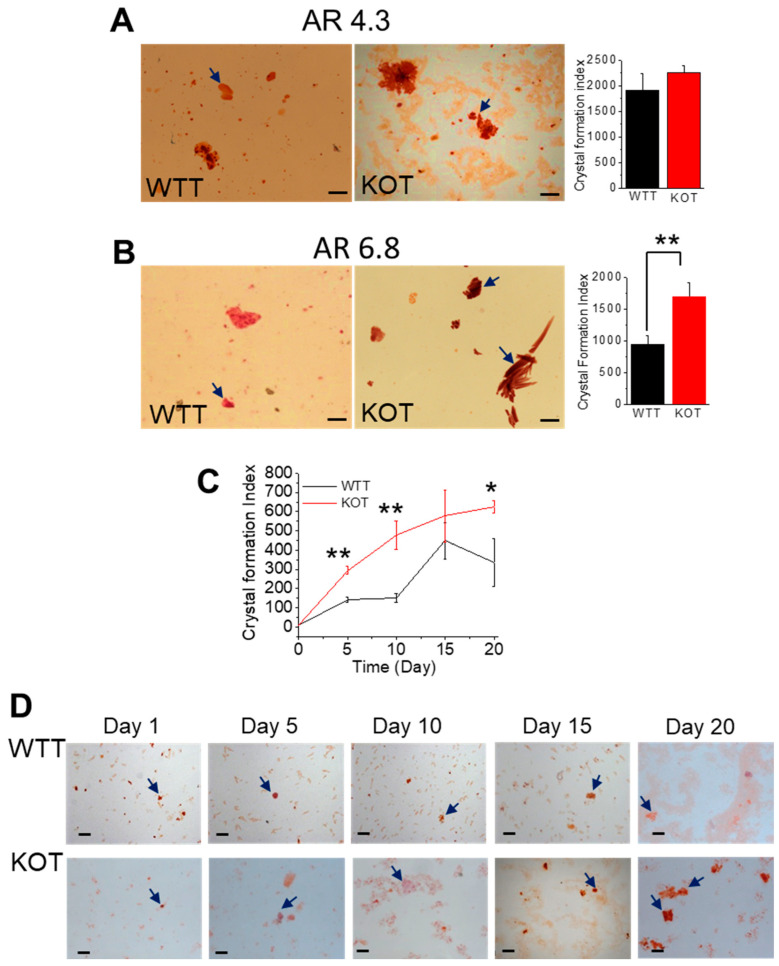
CaP and mixed crystal growth analysis in CaG+ACZ-treated WT (WTT) and TRPC3 KO (KOT) mice urine. Alizarin Red (AR); (**A**) pH 4.3 and (**B**) 6.8 staining were performed in WTT and KOT mice urine to detect CaP or CaOx crystals, respectively. (**C**) Time-lapsed measurements of (**D**) day-to-day WTT and KOT mice urine collection are shown in the depicted images and graph. All graphs in mean + SEM. Urine crystals were collected and measured from *n = 8* mice. *, *p* < 0.05; **, *p* < 0.01.

To assess the stone-forming phenotype of CaG and ACZ-treated TRPC3 KO mice, CaP only and CaP+CaOx urine crystals were measured and compared. Our results show that in CaG and ACZ-treated mice, greater CaP and mixed crystal formation were induced, which was more in the KOT mice compared to the WTT counterparts (Figure 1). We observed a prominent urinary mixed crystal formation in the KOT mice in contrast to their WTT counterparts (Figure 1A–C). Day-to-day CaP crystal measurements were also tracked in both the WTT and KOT mice. Interestingly, by day 5, there was immediate differentiation in the CaP crystal aggregation sizes, with the KOT mice urine crystals remaining greater, until day 15 (Figure 1D). The urine Ca^2+^ and pH measurements were found to be greater in the KOT mice compared to the WTT groups (Figure S1). These results suggest that the initial crystal size increases may be attributed to the rise in PT luminal [Ca^2+^] in the absence of TRPC3, and the extended treatment of CaG and ACZ resulted in a further size increase in both the WTT and KOT mice, while the KOT mice crystal sizes remained larger.

### 2.2. Supplementation of CaG + ACZ Imparts Diffuse Fibrosis and Calcification in TRPC3 KO Kidney Sections

**Figure 2:**
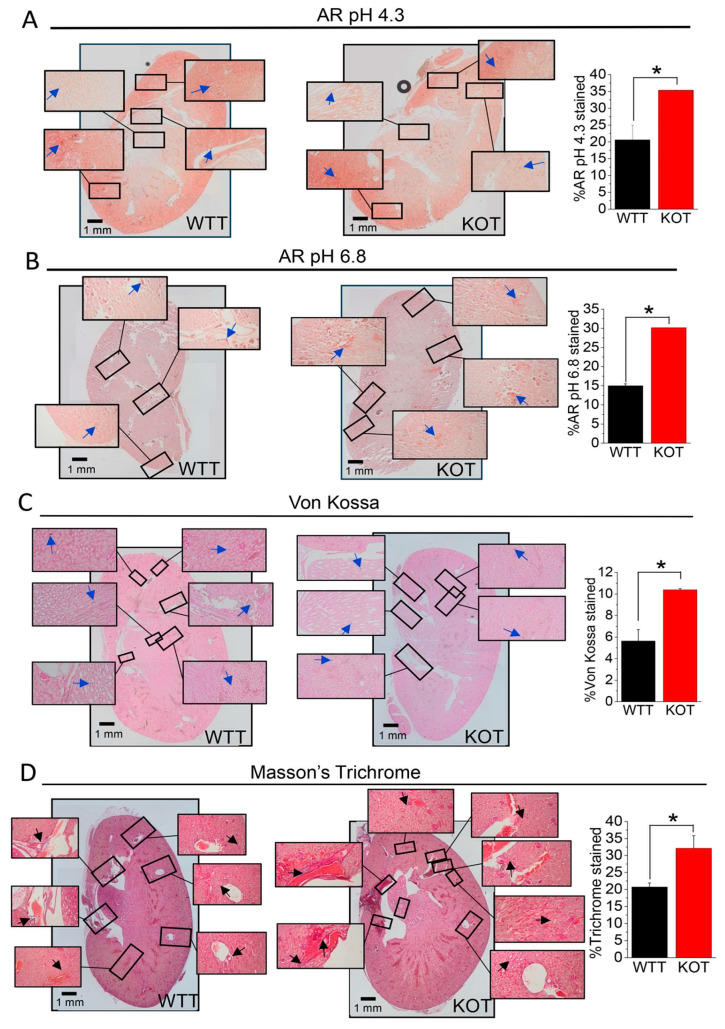
CaG+ACZ-treated WT (WTT) and TRPC3 KO (KOT) kidney section staining to determine fibrosis and calcification. WTT and KOT mice kidney sections were stained with (**A**) Alizarin Red (AR) pH 4.3, (**B**) AR pH 6.8, (**C**) Von Kossa, or (**D**) Massan’s Trichrome staining. Stained kidney sections were retrieved from *n* = 8 mice. Bar diagrams in mean + SEM. *, *p* < 0.05.

CaG+ACZ-treated WT and TRPC3 KO mice kidneys sections were stained with Alizarin Red (AR) pH 4.3 or AR pH 6.8, respectively, to assess CaP and/or CaOx calcification (Figure 2A,B). The treated TRPC3 KO mice sections displayed a greater AR pH 4.3 and AR pH 6.8 stained regions than the treated WT sections in the outer and inner medullary and calyceal areas, implying more CaP and CaOx mixed crystallization in the treated TRPC3 KO mice kidneys (Figure 2A,B). The treated TRPC3 KO mice sections also had a higher percentage of Von Kossa staining, specifically in the outer and inner medullary and calyceal areas, which were substantiated from AR staining (Figure 2A–C). Moreover, we found that fibrosis may be correlated to tissue calcification [14], which has also been supported by Masson’s staining on treated WT and TRPC3 KO mice kidney sections. Interestingly, the fibrotic regions revealed by the staining corresponded with the calcified regions shown by the Von Kossa staining (Figure 2C,D).

### 2.3. CaG + ACZ Treatment Increases SOCE Sensitivity in WTT and KOT Mice Proximal Tubular Cells

**Figure 3:**
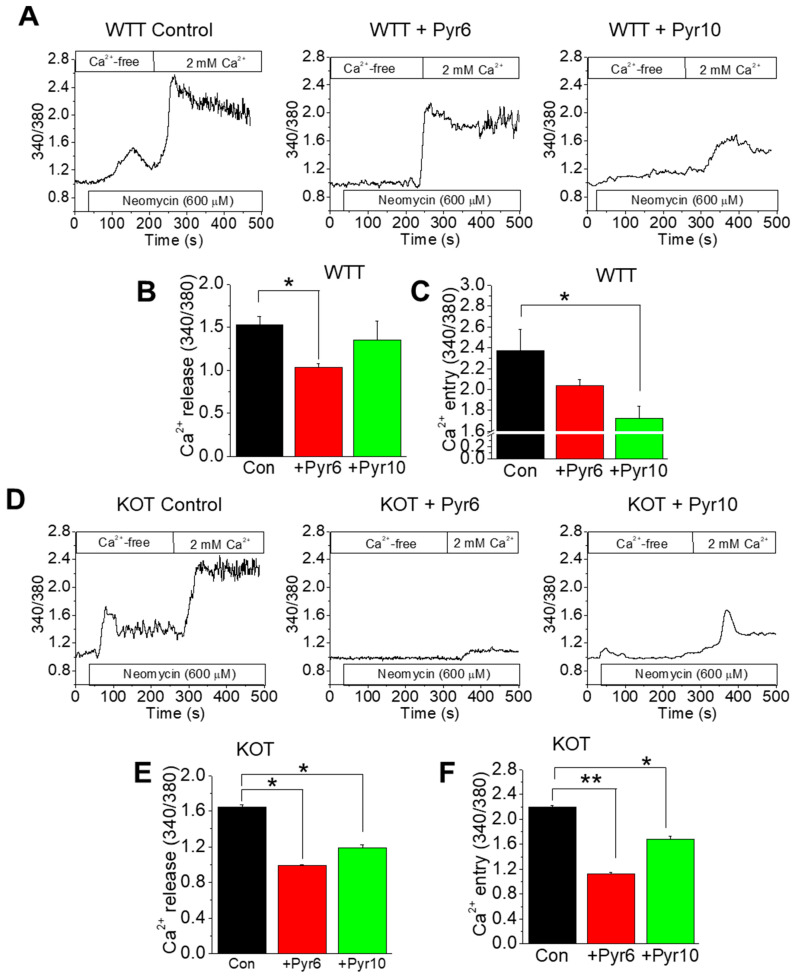
CaG+ACZ-treated TRPC3 KO mice PT cells (KOT) depicted greater ROCE and SOCE compared to their WT counterparts (WTT). Fura-2 ratiometric (340/380 nm) traces of (**A**–**C**) WTT and (**D**–**F**) KOT mice PT cells in a Ca^2+^-free cell bath were obtained. During the experiment, neomycin was implemented and the Ca^2+^ was adjusted to 2 mM. (**B**,**E**) Ca^2+^ release and (**C**,**F**) Ca^2+^ entry components are depicted in bar diagrams as mean + SEM. PT cells were extracted from *n* = 8 mice. *, *p* < 0.05; **, *p* < 0.01.

In our previous studies, the CaSR and TRPC3 were found to be localized within the mice PT cells at the apical brush border [12]. Moreover, CaG or ACZ-only treatment in isolated PT WTT or KOT cells elicited greater Ca^2+^ entry compared to the control counterparts, which was inhibited by Pyr6, suggesting that Ca^2+^ saturation and high pH conditions make the cells susceptible to SOCE [11,13]. Here, we utilized neomycin, a known CaSR activator [15], to assess the Ca^2+^ entry levels in CaG and ACZ-treated WT and KO PT cells to assess the implications of the combined conditions of hypercalciuria and alkalinuria upon the transcellular mechanism of these cells (Figure 3A–F). In the presence of Pyr6 and Pyr10, Ca^2+^ entry in WTT cells was moderately inhibited, suggesting that the Ca^2+^ and pH levels exerted sensitivity changes in the ROCE and SOCE components in these cells (Figure 3A–C). Interestingly, Ca^2+^ entry by KOT PT cells was inhibited even further compared to the WTT counterparts (Figure 3D–F), revealing further susceptibility of KOT PT cells in high Ca^2+^ and high pH conditions.

### 2.4. Enhanced Calcification, Inflammation, Fibrosis, and Apoptosis in TRPC3 KO PT Cells upon CaG+ACZ Treatment

**Figure 4:**
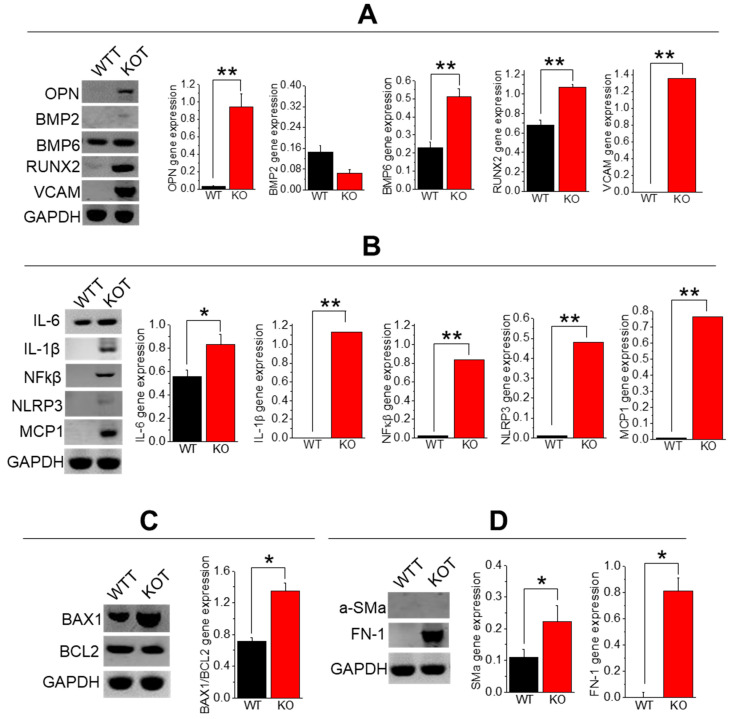
Gene expression profile of CaG + ACZ-treated WT and TRPC3 KO PT cells. Densitometric analysis of (**A**) Calcification genes (OPN, BMP2, BMP6, RUNX2, VCAM), (**B**) inflammation (IL-6, IL-1b, NFκβ, NLRP3, MCP1), (**C**) apoptosis (BAX1, BCL2), and (**D**) fibrosis (a-SMa, FN-1) genes were performed in CaG+ACZ-treated WT and TRPC3 KO mice PT cells. Gene expression was conducted in extracted PT cells from *n* = 4 mice. Bar diagrams are depicted in mean + SEM. *, *p* < 0.05; **, *p* < 0.01. GAPDH was utilized as an internal control gene.

**Figure 5:**
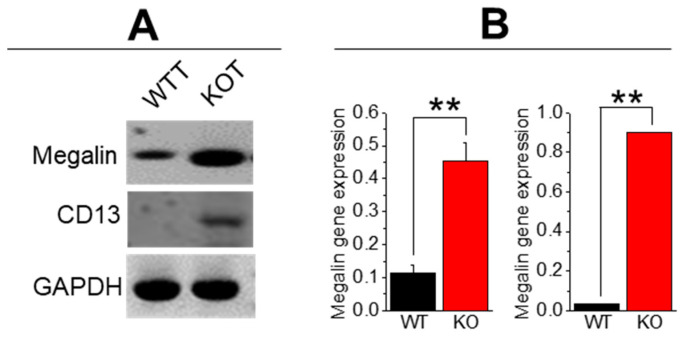
Gene expression profile of CaG + ACZ-treated WT (WTT) and TRPC3 KO (KOT) urine cell debris. (**A**) Gene expression was measured from cellular debris in mice urine from *n = 4* mice Megalin and CD13. (**B**) Densitometric analysis of Megalin and CD13 is depicted in bar diagrams in mean + SEM. **, *p* < 0.01. GAPDH was utilized as an internal control gene.

The TRPC3 ablated mice under either hypercalciuric or alkalinuric conditions showed elevated indicators of inflammation, fibrosis, calcification, and apoptosis compared to the WT and KOT mice [11,13]. In examining gene calcification markers [OPN, BMP-2, BMP-6, RUNX2, VCAM] in the WTT and KOT PT mice cells, OPN, BMP6, RUNX2, and VCAM markers were found to be elevated in the treated KO group compared to the treated WT, which substantiates our findings in Von Kossa, AR pH 4.3, and 6.8 stained mice sections (Figure 2A–C and Figure 4A). In addition, in our profile analysis of inflammatory genes [IL-6, IL-1β, NFkβ, NLRP3, and MCP1], the genes from the treated KO mice were greatly elevated compared to the treated WT mice (Figure 4B). When analyzing the apoptotic marker BAX/BCL2 and fibrotic markers [aSMa and FN-1], the KO cells were increased compared to those in the treated WT cells (Figure 4C,D). These results suggest that the treated WT and KO PT cells reveal a safeguarding effect of TRPC3 against calcification, fibrosis, inflammation, and apoptosis in the presence of higher pH and hypercalciuric conditions in mice. We measured the gene expression in urine cells to assess the sign of cellular damage from the tubular lining, while cellular debris in the urine samples were analyzed for stone crystals. An assessment of urine cells from the WTT and KOT mice showed elevated expression of megalin and CD13, verifying that the PT is one of the main locus points of tissue injury (Figure 5) since megalin [16] and CD13 [17] proteins are exclusively expressed in PT cells.

## 3. Discussion

In our study, moderately hypercalciuric mice (TRPC3 KO) were exposed to higher urinary alkalization and dietary calcium loads, which resulted in enhanced urinary CaP or CaP+CaOx crystal formation compared to their treated WT counterparts. Furthermore, the CaP crystal aggregation sizes in the WTT and KOT mice urine were bifurcated by the 15th day as well as greater mixed crystal formation. Although these results were similarly observed in ACZ or CaG-treated WT and TRPC3 KO mice, the urine crystal-forming phenotypes were much less prominent [11,13]. Other KO mice treatment studies, such as with Npt2a KO mice, experienced increased renal calcium biomineralization after 10 weeks of treatment with high dietary calcium [18]. Importantly, those Npt2a KO mice exhibited amorphous and brushite crystal formations in the control group and dipyramidal CaOx dihydrate in glyoxylate treatment urine groups [18]. In our study, urine samples from the TRPC3 ablated ACZ plus CaG-treated mice groups revealed greater brushite crystal formation with mixed crystals in it. While the phenotype of the WT and TRPC3 KO mice displayed a similar CaP crystal aggregate size with brushite shapes [19], the CaOx crystal sizes were increased in the TRPC3 KO ablated group after twenty days of CaG and ACZ treatment. This may suggest that higher Ca^2+^ supplementation, along with the ablation of TRPC3, may allow greater CaOx to form downstream due to much higher Ca^2+^ delivery. However, whether more or less CaP or CaOx is formed throughout the nephron during the treatment is uncertain, unless the entire pathway is tracked within an in vivo model. Our results can be explained by the fact that the urine pH may be intrinsically high in some people who are thereby predisposed to CaP stones [4]. Other studies found that increasing pH has been shown to enhance Ca^2+^ uptake in transient receptor potential vanilloid 5 channel, and reducing calciuria can lead to calcium retention from a negative calcium balance [20]. Furthermore, the luminal pH at the end of the proximal straight tubule is approximately 6.7–6.8 [21], which also means that the PT plays an essential role in net acid excretion. This study demonstrates the impact of calciuria and urine pH on crystal formation in the PT. Previous studies have shown that urinary supersaturation with respect to CaOx and CaP is generally achieved at different locations in the renal tubules [22]. Importantly, we have demonstrated that tubular CaP crystals can form mixed with CaOx [11,12,13], and others have shown CaOx crystals in ethylene glycol-induced rats [22]. The CaP crystals formed in our study can be termed poorly crystalline with a large amount of substitution within the unit cell, with amorphous crystalline associated with other crystalline materials [19].

Since PT cells engage in transcellular Ca^2+^ transport, we found elevated SOCE Ca^2+^ entry in treated TRPC3 KO cells using SOCE (Pyr6) and ROCE (Pyr10) inhibitors. Ca^2+^ entry by WTT cells was moderately inhibited, suggesting that the Ca^2+^ and pH levels exerted changes in the ROCE and SOCE components in these cells. The SOCE pathway has been shown to promote deleterious conditions within the PT cell when modified from physiological conditions due to crystal induction or contaminants [23]. In both cases, we found that CaG-only and ACZ-only treatments of PT cells modulated the Ca^2+^ entry by augmenting in TRPC3 KO compared to the WT [11,13]. Similarly, we found that treated TRPC3 KO mice PT cells utilized greater SOCE compared to treated WT mice. Furthermore, in our previous work, we showed that under normal (physiological) conditions, the majority of the Ca^2+^ entry/transport in the PT cells was through ROCE and that the amount was diminished compared to the SOCE in TRPC3 ablated cells [12,23]. The implication of the resulting greater SOCE in the present study could be leading to more PT cell damage due to the treatment condition (ACZ + CaG), which can aggravate the process of stone formation [11,13].

The rise in urine pH can be caused by renal damage from stone crystals or procedures or treatments for stones [24]. If this is the case, it is possible that the treatments producing greater amounts of local inflammation could result in a higher likelihood of acidic blood pH that would result in elevated urinary pH and greater local crystal deposition in the future [25]. This may also offer a possible explanation for the lack of a response in some patients’ experiences with urinary alkalinization for calcium stone treatment. We have shown that ACZ + CaG-treated KOT mice experienced greater renal calcinosis compared to WTT mice. Diffuse fibrotic regions were observed in the KOT mice compared to the WTT mice, with inflammation, calcification, and apoptotic gene expression increased in the KOT mice compared to the WTT mice, which was corroborated in our previous findings [11,13]. The mechanistic link was provided between hypercalciuric conditions, alkalinuric conditions, and the exacerbation of renal fibrosis and inflammation, particularly in the absence of TRPC3. Research indicates that the uptake of crystals initiates an innate immune response through the release of IL-1β, altering the understanding of the development of disorders related to crystals [26]. The elevated expression of fibrotic markers, specifically TGF-β1, FN-1, and SMα, also establishes a connection between hypercalciuria, alkalinuria, and renal fibrosis [27]. The simultaneous upregulation of both fibrotic and inflammatory genes in the treated groups suggests that hypercalciuric stress might have triggered the immune axis to play a protective role in renal fibrosis [28]. Interestingly, NLRP3 appears to have a protective effect on renal fibrosis, as evidenced by conditions where it is knocked down or inhibited [29]. The activation of NLRP3, similar to our findings, has been observed in dendritic cells, although it is independent of the rise in [Ca^2+^]_i_ [30]. This activation process could potentially offset the increase in renal fibrosis under hypercalciuric and alkalinuric conditions, but this will depend on the outcomes of future studies in this area.

We utilized CaG to examine how prolonged hypercalciuric stress could be implicated in mixed calcium stone formation and cellular injury [13], and to assess the effects of alkalinuria. The reason we chose to use both treatments in this study was to find an aggravated mixed stone-forming phenotype in our mice model and examine the crystal formation in the resultant phenotype of the mice. We show in this study that more mixed crystal formations were found with greater CaP and CaOx formations within the urine of TRPC3 KO mice compared to that of treated WT mice (Figure 1). The results were different in the ACZ-only treated TRPC3 KO and WT mice, where there was no difference in mixed crystal formation, but there was more CaP crystal formation in the urine [11]. However, the pH was not measured at the tubular level. Instead, we assessed the urine pH and that there may be a possibility of modification due to further distal tubular effects. Nevertheless, the implications of these results mean that different treatment options may be suitable for each of these conditions (ACZ, CaG, and ACZ+CaG). Dietary intake could spontaneously change the pH of the urine, which could affect the treatment of urolithiasis and kidney stones. Higher meat intake with less intake of fruits and vegetables has been correlated with lower urinary pH, whereas lower meat intake with higher fruit and vegetable intake has been correlated with elevated urine pH [31], and these dietary effects were examined to change the pH of the urine within 3 days of switching diets [32]. However, the pH changes in these mice resulted from mechanistic changes, i.e., inhibition of the carbonic anhydrase by ACZ, and were not due to alkali loading.

Carbonic anhydrase inhibitors like ACZ and Topiramate are commonly used as medication for the treatment of heart failure, seizure disorders, migraine headaches, glaucoma, etc. [33]; but any prior history of nephrolithiasis should be considered in the risk/benefit ratio when using this family of medications. Furthermore, the age of the mice may be a factor in the progression of urine crystal development for knockout mice [34]. In other mice studies, such as Npt2a^−/−^ mice with hypercalciuric and hyperphosphaturic, intratubular and interstitial deposits of poorly crystalline biological apatite were detected across a broad age range (5 days to 12 months) [34]. Furthermore, the number of interstitial deposits increases with age and most of them are present after the age of 5 months [34]. However, it may be out of the scope of our present study, and the mice we used are of similar adult age. We and others have also shown that TRPC3 ablation relates to Ca^2+^ wasting, which is independent of PTH regulation [35]. We acknowledge that considering this is an animal study, there are limitations to the translational aspects of the findings. However, our previous studies have shown that the C57BL/6 mice revealed regions of anti-TRPC3 stained in the apical localization of the PT, while several other studies have also shown the existence of TRPC3 through gene and protein quantification means [11,13,36,37]. Furthermore, interspecies comparisons have been possible due to mice and human PT expression of TRPC3 in the human PT appearing to have similarly analogous TRPC3 prevalence in the murine PT [38]. Renal stone formation is a complex process involving primary and secondary crystal nucleation, aggregation, and growth to the point that the overgrowth can stick to the tubular wall to stop the flow of urine. In our mice model, we captured the maximum urinary crystal formation as a “pathophysiological snapshot” although we did not see a complete stop of urinary flow. Our preliminary characterization of those crystals enables us to present the size, category, and severity of the effect of a higher pH of tubular fluid and/or urine along with the escalation of tubular [Ca^2+^]. Nevertheless, further precise crystal analysis, such as infra-red spectrometry or Raman analysis, may be needed to elucidate the nature of those crystals.

## 4. Methods and Materials

### 4.1. Animals

All the animals used in this study were approved as part of a protocol designed in accordance with the Guiding Principles in the Care and Use of Animals, approved by the Institutional Animal Care and Use Committee (IACUC) and the Research and Development Committee of DC Veterans Affairs Medical Center. Wild Type (WT) and TRPC3 KO (−/−) mice purchased from the Jackson Laboratory (Bar Harbor, ME, USA) were maintained and crossed as described previously [12]. We alkalinized the tubular pH in TRPC3−/− mice by oral ACZ (0.08%) and induced hypercalciuria using 2% CaG [11,13]. To avoid anhedonia, 2% sucrose was used with ACZ and CaG supplementation in WT mice, denoted as the WTT group, and TRPC3 KO mice, denoted as the KOT group. The WT and TRPC3 KO mice treated with 2% sucrose alone were used as respective controls to their CaG- and ACZ-treated counterparts. All the mice were housed in individually ventilated cages under controlled environmental conditions (12 h light/12 h dark cycle, room temperature 21–23 °C, humidity 50–60%) with free access to standard food pellets and water. The ages of the mice ranged from 6 to 12 weeks. Data on calcium, food, and water intake were recorded to determine any differences in dietary intake (calcium, food, and water) between the treated and untreated groups.

### 4.2. Chemicals

CaG, ACZ, neomycin, Pyr6, and Pyr10 were purchased from Sigma–Aldrich (St. Louis, MO, USA). Dulbecco’s Modified Eagle Medium (DMEM) media, fetal bovine serum (FBS), antibiotics (penicillin and streptomycin), glutamine, and Fura-2-acetoxymethyl ester (Fura-2-AM) were purchased from Invitrogen (Carlsbad, CA, USA). All the chemicals were of analytical grade.

### 4.3. Isolation and Primary Culture of PT Cells

PT cells from CaG and ACZ untreated or treated mice were isolated and cultured as previously described [11,37]. The kidneys were decapsulated and then the cortical tissues were immediately extracted and washed with a cold external solution containing 0.02% soybean trypsin inhibitor and 0.1% bovine serum albumin. The cortical tissues were used to obtain PT cells through an enzymatic digestion buffer containing 1% Worthington collagenase Type IV and 0.25% soybean trypsin inhibitor. The cells were then passed through a nylon mesh (pore size 70 µm) filter and then resuspended by centrifugation at ~27,000× *g* for 10 min at 4 °C. The cells were washed with media (DMEM containing 10% FBS, with 2 mmol/L glutamine, 100 IU/mL penicillin, 100 µg/mL streptomycin, 5 µg/mL insulin, 5 × 10^−8^ mol/L hydrocortisone, 5 µg/mL transferring, 2 mmol/L butyrate, 2 mmol/L alanine, and 2 mmol/L lactate supplementation) and were immediately cultured as described [12] for Ca^2+^ imaging experiments.

### 4.4. Fura-2 [Ca^2+^]_o_ Measurements

[Ca^2+^]_i_ measurements were conducted in Fura-2 loaded PT cells as described previously [12]. These cells were then placed in a microincubator with a temperature of 37 °C and a gaseous mixture of 95% air and 5% CO_2_. Images of the cells were taken using an IX81 motorized inverted microscope equipped with an IX2-UCB control box (Olympus USA, Center Valley, PA, USA) and were fed into a C9100-02 electron multiplier CCD camera with an AC adaptor A3472-07 (Hamamatsu, Bridgewater, NJ, USA). The fluorescence emitted by Fura-2 was excited at wavelengths of 340 nm and 380 nm and the emission was collected at a wavelength of 500 nm. The PT cells were brought into focus by a differential interference contrast (DIC) channel. These measurements were digitally processed using the 3i SlideBook version 5.0 microscopy software (Intelligent Imaging Innovations, Denver, CO, USA). The time-lapse was set at 800 time points at 1 s intervals in an average of 50–150 cells for each experiment, where regions of interest were selected (background fluorescence automatically subtracted prior to 340/380 ratio calculation and graphing). The analysis was performed offline using Slidebook™ software (Olympus, Center Valley, PA, USA). The Ca^2+^ release and Ca^2+^ entry in the ratiometric (340/380) Fura-2 traces for Figure 3 were distinguished by the first and second troughs within the Ca^2+^ transient. Neomycin (agonist) was used in a Ca^2+^-free media to elicit the Ca^2+^ release and then the readjustment of the physiological Ca^2+^ condition (2 mM) in the cell bath was implemented to examine the Ca^2+^ entry response.

### 4.5. Alizarin Red Staining of Urine Crystals

Samples of 24 h mice urine were collected from mice in metabolic cages and immediately centrifuged to collect the pellets for crystal analysis. AR staining was performed on these pellets to detect the presence of CaP and/or CaOx crystals, as described previously [12,19]. The urine was briefly centrifuged, and the crystals and cells were subsequently collected. Equal volumes of AR pH 4.3 (stains CaP crystals) or pH 6.8 (stains both CaOx and CaP/CaOx mixed crystals) were added to the crystals, which were then incubated at 37 ºC for 30 min. Afterwards, the supernatants were removed, and those crystal pellets were mounted on slides to capture images of the stained crystals using light microscopy (Zeiss Axio Observer.Z1 Microscope). The stained images were quantified using ImageJ, as previously described [14].

### 4.6. RT-PCR from Isolated PT Cells

RNA was extracted from isolated mice PT cells from mice kidneys using TRIzol, as previously described [12,13]. Mice urine samples were centrifuged to collect the urinary debris, and the urine cells were further separated for RNA extraction [39]. The RNA was then separated from the aqueous phase after chloroform addition and precipitated using 2-propanol followed by 75% ethanol. The precipitated RNA was air-dried and resuspended in DEPC-treated water. The dissolved RNA was further purified using a DNase I Application Grade Kit (Sigma–Aldrich, St. Louis, MO, USA) according to the manufacturer’s instructions. The purified RNA was quantified using a nanodrop spectrophotometer (ThermoFisher Scientific, Waltham, MA, USA) and used for cDNA synthesis. The cDNA was prepared using a GoScript™ Reverse Transcription System (Promega, Madison, WI, USA) according to the manufacturer’s instructions. The PCR Master mix was prepared using the GoTaq Green Master Mix (Promega, Madison, WI, USA) according to the manufacturer’s protocol. In total, 15 μL of Master mix was added to 5 μL of cDNA template at a 1:10 dilution. A list of primer sequences used in this study is shown in Table 1. The thermocycling parameters were as follows: initial denaturation at 95 °C for 3 min, subsequent PCR cycles (x35) of denaturation at 95 °C for 30 s, annealing at 55 °C for 30 s, extension at 72 °C for 45 s, and final elongation at 72 °C for 5 min.

### 4.7. Histochemistry of Kidney Sections

Kidneys collected from the mice after euthanasia were immediately fixed in 10% formalin solution for 24h and then dehydrated in graded concentrations of ethanol and then embedded in paraffin. Mice tissue sections (∼5–7 µm) were prepared from whole kidney tissue paraffin blocks. Histochemistries were performed on paraffin sections of treated mice kidneys, as described previously [11,12,13]. Sections were stained with H&E, Massan, Von Kossa, or AR pH 4.3 or pH 6.8, as described previously [11,12,13,40]. All the images were quantified from n = 8 kidneys using ImageJ software. Measured areas of AR pH 4.3 or 6.8, Von Kossa, and Masson’s Trichrome staining were performed using either the manual hand selection measurement or the color thresholding selection of color changes pertaining to a positive indication of tissue fibrosis, according to the individual stain [11,12,13,14].

### 4.8. Statistical Analysis

To assess the differences in birefringent particles, four-factor repeated measures ANOVAs were performed using the general linear model for groups of unequal sample size, with independent factors of gender, feed treatment, and TRPC3 status and repeated factor of kidney region. The sample size of each experiment was numbered from *n* = 4–8. When warranted by a significant F-statistic, ANOVA followed by *post hoc* Tukey tests was used to assess pairwise comparisons between individual mice with different genders, treatments, and genetic knockout conditions, by kidney region. The significance was set at *p* < 0.05 for all the tests. In other experiments, student’s two-tailed t tests were performed between the groups.

## Figures and Tables

**Table 1:** PCR primer information.

Primer	Sequence (Sense, Antisense)	Product Size (bp)
GAPDH	5′-ACTCCACTCACGGCAAATTC-3′ 5′-TCTCCATGGTGGTGAAGACA-3′	171
OPN	5′-GAGGAAACCAGCCAAGGACTAA-3′ 5′-TCCAGACTTGGTTCATCCAGC-3′	695
BMP2	5′-TGGAAGTGGCCCATTTAGAG-3′ 5′-TGACGCTTTTCTCGTTTGTG-3′	166
BMP6	5′-CCCGCCCGGAGTAGTTTTAGC-3′ 5′-AGTGCCCTTCTCCCCTCCATT-3′	168
RUNX2	5′-CGGCCCTCCCTGAACTCT-3′ 5′-TGCCTGCCTGGGATCTGTA-3′	330
VCAM	5′-CCCAAGGATCCAGAGATTCA-3′ 5′-TAAGGTGAGGGTGGCATTTC-3′	489
IL-6	5′-TGGAGTCACAGAAGGAGTGGCTAA-3′ 5′-TCTGACCACAGTGAGGAATGTCCA-3′	155
IL-1β	5′-TCCATGAGCTTTGTACAAGGA-3′ 5′-AGCCCATACTTTAGGAAGACA-3′	343
NFκβ	5′-GTGGAGGCATGTTCGGTAGT-3′ 5′-AGCTGCAGAGCCTTCTCAAG-3′	367
NLRP3	5′-TGGGTTCTGGTCAGACACGAG-3′ 5′-GTCATTCCACTCTGGCTGGT-3′	486
MCP1	5′-AGGTGTCCCAAAGAAGCTGT-3′ 5′-AAGACCTTAGGGCAGATGCA-3′	163
BAX1	5′-GGAGACACCTGAGCTGACCT-3′ 5′-CTCAGCCCATCTTCTTCCAG-3′	510
BCL2	5′-TCGTCGCTACCGTCGTGACTTCG-′ 5′-AGAGTCCGGTTCAGGTACTCAGTC-3′	242
Megalin	5-GTTCGGGTTGATGTTCTGGA-3′ 5′-ACTTGGGTAAGCCAGGGGTT-3′	369
aSMa	5′-AGATTGTCCGTGACATCAAGG -3′ 5′-TTGTGTGCTAGAGGCAGAGC-3′	538
FN-1	5′-AATCCGGGAGCTTTTCCCTG-3′ 5′-GAGCTTCCTGTCCTGTCTTCT-3′	971
mCD13	5′-TCACAGTGATAACGGGAAAGCCCA-3′ 5′-ATAAGCTCCGTCTCAGCCAATGGT-3′	799

## Data Availability

The data that support the findings of this study are available from the corresponding author upon reasonable request.

## Supplementary Materials

The following supporting information can be downloaded at: https://www.mdpi.com/article/10.3390/ijms25094787/s1.
