# Supplementary material for: The Rise in Tubular pH during Hypercalciuria Exacerbates Calcium Stone Formation
Source: Int J Mol Sci. 2024 Apr 27;25(9):4787. doi: 10.3390/ijms25094787 (PMC11084476; doi:10.3390/ijms25094787)
Supplement: Supplementary file 1 [file ijms-25-04787-s001.zip › ijms-2941342-supplementary.pdf]

SUPPLEMENTAL MATERIALS

The rise in tubular pH during hypercalciuria exacerbates calcium stone formation

Farai Gombedza [1], Samuel Shin [1,2], Jaclyn Sadiua [1], George B. Stackhouse [3] and Bidhan C. Bandyopadhyay [1,2,4*]

[1] Calcium Signaling Laboratory, Research Service, Veterans Affairs Medical Center, 50 Irving Street, NW, Washington, DC 20422, USA
[2] Department of Biomedical Engineering, The Catholic University of America, 620 Michigan Avenue NE, Washington DC, 20064, USA.
[3] Urology Service, Veterans Affairs Medical Center, 50 Irving Street, NW, Washington, DC 20422, USA
[4]Division of Renal Diseases & Hypertension, Department of Medicine, The George Washington University, Washington DC, 20037, USA

Running title: Rise in tubular Ca^{2+} and pH induces Calcium Nephrolithiasis

Keywords: hypercalciuria; renal tubular pH; proximal tubule; oxidative stress; inflammation; fibrosis; apoptosis; calcium nephrolithiasis

[*]**Corresponding author**: Bidhan C. Bandyopadhyay, PhD. Chief, Calcium Signaling Laboratory, 151 Research Service, Veterans Affairs Medical Center, 50 Irving Street, NW, Washington, DC 20422, Phone: (202) 745-8622, Fax: (202) 462-2006; E-mail: bidhan.bandyopadhyay@va.gov

Supplemental Figure

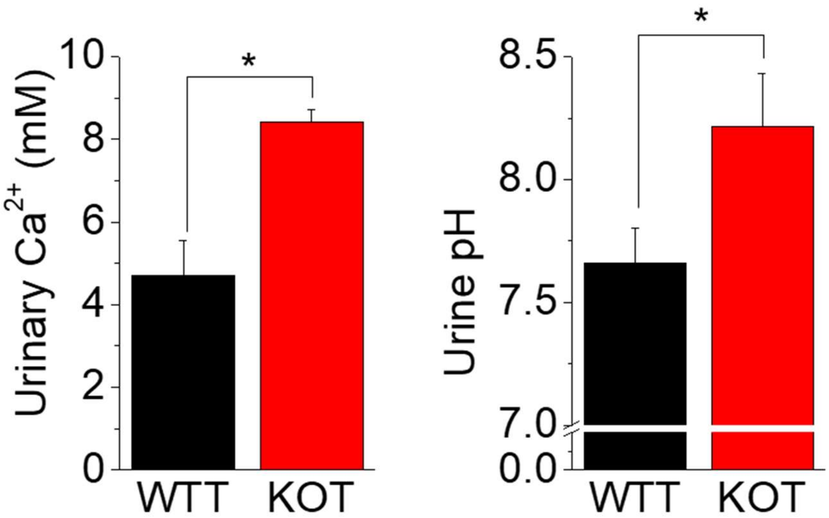

Figure S1. Urinary Ca^{2+} and pH measurements of mice following 2% calcium gluconate plus 0.08% acetazolamide treatments. Bar diagrams show significant changes between the treated group (WTT and KOT). Representative bar graphs are plotted from means ±SEM of 4 separate experiments. *, $p < 0.05$.
